# Physicians’ Lived Experience of Breaking Bad News in Clinical Practice: Five Essentials of a Relational Process

**DOI:** 10.1177/10497323231197534

**Published:** 2023-10-04

**Authors:** Mattias Tranberg, Eva M. Brodin

**Affiliations:** 1 Department of Clinical Sciences Lund, 59568 Lund University, Lund, Sweden; 2 The Institute for Palliative Care at Lund University and Region Skåne, Lund, Sweden; 3 Department of Educational Sciences, 59568 Lund University, Lund, Sweden; 4 Centre for Higher and Adult Education (CHAE), Stellenbosch University, Stellenbosch, South Africa

**Keywords:** communication, phenomenology, physicians, patients, serious illness

## Abstract

The purpose of this study was to develop deeper knowledge about physicians’ lived experiences of breaking bad news by identifying their common meanings and interrelatedness along with their potential alignment with process-oriented and relational aspects. Based on the methodology of descriptive phenomenology, in-depth interviews were conducted with 22 physicians from a wide variety of specialties. The participants were invited to freely reflect upon their experiences of breaking bad news by describing situations that had worked well and less well. Results showed that breaking bad news was fundamentally experienced as a *relational process* constituted by the five essentials of *Becoming the bad messenger*, *Expecting the unpredictable*, *Being on stage*, *Professionally managing hope*, and *Mindfulness of the emotional relationship.* In line with recent research, this study confirms that clinical communication involves much more than just delivering the message. However, it also contributes to existing knowledge by focusing on the phenomenology of physicians’ experiences, which enables deeper understanding of the medical profession and the relational process of breaking bad news. As such, our findings are important to enable broader learning in, for example, medical education and continuing courses for clinical staff.

## Introduction

If individuals have a choice, they generally prefer to offer good news rather than bad, which is known as the minimizing unpleasant message (MUM) effect (Dibble & Levine, 2010). Nonetheless, physicians frequently need to tell patients about serious diagnoses and prognoses; that is, they need to *break bad news*, sometimes even before a patient experiences any symptoms (Caballero et al., 2014; Rajan et al., 2015; Whitaker, 2020). In severe cases, they must also share new discouraging findings and unfavorable developments that cannot be avoided through treatment (Alshami et al., 2020). In such situations, physicians often minimize an unpleasant message by lessening the gravity of the bad news—for fear that a patient will feel bereaved of hope and become depressed (Berkey et al., 2018).

Findings from multiple studies in various settings show that recipients tend to blame the messengers, find them less empathic (Tranberg et al., 2023), and ascribe to them malign intent even when the status quo is out of the messengers’ control (John et al., 2019). Even though physicians are expected to encourage and allow their recipients to express themselves within reasonable limits (Lee et al., 2002), physicians themselves become vulnerable in these situations, and several studies have shown that they therefore suffer physiological stress reactions (Studer et al., 2017), along with feelings of anxiety, guilt, exhaustion, failure, and frustration (Bousquet et al., 2015). Still, most physicians perceive their communication with their patients as very important, although only a few feel that they have received sufficient training to communicate effectively (Monden et al., 2016).

### Breaking Bad News as Communication Skills

Some decades ago, Buckman (1984) called for research and education related to breaking bad news, that is, communicating “any information that has a serious and adverse effect on an individual’s view of his future” (Buckman, 1984, p. 1597). Since then protocols have been developed for such purposes in, for example, the United Kingdom (Maguire & Faulkner, 1988) and the United States (Baile et al., 2000), along with consensus guidelines for communication (Gilligan et al., 2017). Also, courses in communication skills, such as *VitalTalk* and *Comskil*, have provided clinicians with a structure to follow when disclosing new information and practice concerning how to explore and support emotional cues with simulated patients (Johnston & Beckman, 2019). Furthermore, during the past decade, health care practitioners have learned techniques from improvisational theater in *Med-improv training* to become more present and adaptive in breaking bad news (Kukora et al., 2020). More recently, comprehensive initiatives such as the *Serious Illness Care Program* were launched, involving clinician communication skills training, preparation of patients and families, a structured guide for conversations, and tailored electronic medical record modules for documentation (Bernacki et al., 2015).

Taken together, such educational and quality improvement measures have enhanced the conditions for breaking bad news in some respects. Randomized controlled trials show that health care practitioners who have participated in communication skills training are more likely to show empathy toward their patients than those who lack such training, although there is no evidence yet that such courses have any effect on physician burnout, patient satisfaction, or patient perception of communication skills (Moore et al., 2018). Moreover, physicians now initiate conversations about serious illness earlier with their patients, and they also continue these conversations throughout the clinical process (Back et al., 2009). This procedure has led to reduced anxiety and depression in some patients (Karim et al., 2022)—yet, the clinicians themselves still find it hard to discuss patients’ prognoses and respond to their emotions (Geerse et al., 2019). Against this background, it is justified to question whether breaking bad news is all about *delivering* emotionally proper communication.

### Toward a Process-Oriented Approach

In clinical practice, breaking bad news implies to enter another person’s life at a very vulnerable moment (Applbaum, 2017). Physicians meet patients who are usually emotionally affected by their situation and sometimes also have cognitive difficulties with understanding the message, for example, about their options in treating cancer (Reyna et al., 2015). Hitherto, knowledge about breaking bad news has developed from qualitative studies of how physicians and patients experience such conversations. Based on their meta-synthesis of 40 qualitative studies including data from over 600 oncologists, Bousquet et al. (2015) found that oncologists’ experienced difficulty in breaking bad news was either related to external factors, such as the health care system and cultural differences, or to communication with the patient. Among other things, they had to balance their information to the patient and consider different communication styles along with concern about the patient’s emotions. Bousquet et al. (2015) nonetheless emphasized that most of the studies had focused on specific aspects of breaking bad news rather than on the entire process.

However, Miller et al. (2022) concluded from their meta-synthesis of studies from palliative care that delivering bad news appears to be a process indeed—and very much a circular one—since the same conversation between the physician and the patient is repeated as their relationship develops. Therefore, Miller et al. (2022) suggested that physicians should adopt therapeutic relationships to their patients which involve care, trust, and continuous communication. Similarly, Matthew et al. (2019) found in their meta-synthesis of patients’ and patients’ family members’ experiences that receiving bad news was not limited to a single moment but that it involved three prolonged phases: *preparation*, *delivery*, and *adjusting and coping*. In the same study, Matthew et al. (2019) also found that it was important for patients to receive bad news from someone who had built a good relationship with them by respecting their preferences for what and how much information they wanted. In cases where a good relationship had not yet developed because of time constraints, their first impression of the physician became even more important. Furthermore, through follow-up appointments with the same physician, the patients did not associate the initial bad message with abandonment. Instead, the continuous relationship increased the patients’ ability to cope with their diseases (Matthews et al., 2019).

### Toward a Relational Approach

In parallel with the recent process-oriented approach, a relational approach has also received increased attention in the literature. Within this movement, the established biomedical model (focusing on the disease) is criticized in favor of a biopsychological model (Engel, 1980), where the patient is essentially understood as a human being. This approach acknowledges the interplay between biological, psychological, and social factors in relation to health and illness, highlighting the significance of addressing various facets of an individual’s well-being. Holding that health care is essentially about caring for human relationships (Soklaridis et al., 2016), this movement implies a change in the clinician’s attitude to patients from empathy to *compassion*, which is more value-driven and emphasizes the relational aspects of breaking bad news. In health care, compassion has been defined as “a virtuous and intentional response to know a person, to discern their needs and ameliorate their suffering through relational understanding and action” (Sinclair et al., 2021, p. 1057). There is also wider definition “a sensitivity to suffering *in self and others* with a commitment to try to alleviate and prevent it” (Gilbert & Simos, 2022, p. 10), which is used in other contexts, such as compassion-focused therapy.

Taking the relational movement one step further, Berger and Ribeiro Miller (2022) directed criticism at the notion of breaking bad news per se and instead suggested the expression “sharing serious information.” By this it is meant that there is a conceptual shift from focusing on clinician-based to relationship-centered care, in which the latter warrants shared decision-making with patients as well as less emotionally charged language. Without contesting the relational approach of “sharing serious information,” we will nonetheless continue to use the notion of “breaking bad news” in this article, since it prevailed when the study was conducted. Also, “breaking bad news” is still an established concept in both research and practice.

### Toward a Deeper Understanding of Breaking Bad News

Until now, research about breaking bad news has mainly focused on best practices and guides to enhance the technical skills of health care practitioners in giving information and responding to patient emotions. Even though the complex and relational process of breaking bad news has recently been highlighted in studies on best clinical practice (Davies et al., 2017), the primary focus has been on improving practice per se, rather than on developing deeper understanding of physicians’ own experiences of breaking bad news. Thus, it is not clear yet *how* the relational process is *constitutive* for physicians’ experiences of breaking bad news—if at all.

Assuming that breaking bad news in clinical practice involves more than transferring information and responding to emotions, the purpose of this study was to develop deeper knowledge about physicians’ lived experience of breaking bad news. Focus was directed toward finding the common meanings and their interrelatedness within the physicians’ described experiences, that is, the essential structure of the phenomenon of breaking bad news, and to reveal how this structure is potentially aligned with process-oriented and relational aspects. Against this background, our study aimed to answer the following research questions:

• What are the common meanings of breaking bad news in clinical practice as experienced by physicians?
• How are these meanings interrelated to each other over time?
• In what ways do relational aspects structure the physicians’ overall experience of breaking bad news (or not)?

## Methodology

This study is based on descriptive phenomenology according to Giorgi’s (2009) methodological approach. Ontologically, this means that we understand the physicians’ lifeworld, that is, their clinical practice, as being based on both their subjective and intersubjective experiences, while “experience” here refers to any thoughts that physicians associate with breaking bad news to their patients. Epistemologically, we are interested in the meanings of *what* is perceived, as well as *how* these meanings are temporarily and spatially constituted in relation to each other. Focus is on finding the common meaning constitution across various experiences, that is, to disclose the intrinsic and general *structure* of breaking bad news.

### Participants

Inclusion criteria encompassed working physicians with experience of breaking bad news. An email was sent to forty-seven physicians who had signed up for a communication course, of which a total of twenty-two physicians (sixteen women and six men) consented to participate in the study. Among these, fourteen had signed themselves up for the course and eight had been prompted to participate by their supervisors. The participants were from 30 to 55 years of age and were either specialists or at the end of their residency. The specialties included cardiology, ear–nose–head–neck, geriatrics, infectious diseases, internal medicine, neurology, neurosurgery, oncology, ophthalmology, pediatrics, palliative medicine, psychiatry, and rheumatology.

### Data Collection

Data were collected before the physicians started the communication courses. In-depth interviews, ranging for about 1 hour, were conducted in Swedish by the first author with each of the twenty-two participants (labelled P1‒P22). To enable phenomenologically rich descriptions of their lived experiences (Dahlberg et al., 2008), the participants were invited to reflect upon their experiences of breaking bad news by freely describing (1) a situation where they had broken bad news to a patient or a patient’s family member, (2) a situation that had worked well, and (3) a situation that had worked less well. Follow-up questions were asked to deepen the understanding of how they had experienced the situation, for example, “How did you react/feel, think about…?”. All interviews were transcribed verbatim before analysis. Quotes have been translated into English in the results.

### Data Analysis

Following Giorgi’s (2009) phenomenological approach, the first author initially performed the phenomenological reduction through exhaustive and reiterated reflection and writing about all his presupposed knowledge and feelings about the topic. In the phase of analyzing the interviews, all transcripts were first read through to get an overall sense of their content. Thereafter, each transcript was analyzed independently by dividing it into different units of meaning, that is, where a *shift of meaning* could be noticed in the physician’s described experience. These units were then transformed into their *psychological* meanings by the first author employing *free imaginative variation*, focusing on the physicians’ integrated behavior, emotions, and potential relational concerns. Free imaginative variation is a reflective process where the researcher without being bound by strict rules or constraints creatively removes or changes elements of the text to determine if this removal or change transforms the description in an essential way (Giorgi, 2007).

In the next step, the structure of the phenomenon was outlined in collaboration between the authors. In gestalt terms, a structure is comprehended as a unified entity, but one that is exclusively comprised of essential parts (Englander & Morley, 2023). Again, using free imaginative variation, the authors reflected jointly upon what aspects of psychological meaning appeared to be necessary for the phenomenon of breaking bad news to be intact, while redundant meanings were removed. Here, free imaginative variation was executed by changing or removing some aspects (e.g., hope) to see whether the phenomenon of breaking bad news then collapsed. When the removal of an aspect leads to the collapse of the phenomenon, it signified that the aspect was essential. Conversely, if modifying a particular aspect had minimal impact on what was being presented, it indicated that the aspect was non-essential. In cases where a particular unit turned out to be necessary, it was retained; otherwise, it was removed to further condense the meaning.

Finally, the remaining units of meaning were synthesized into a coherent description of the essential structure of the phenomenon.

While some methods in phenomenology require researchers to ask for participant validation of the results (Beck, 2021), Giorgi (2006) argues against such procedures since the researcher and participant inevitably have different perspectives: for example, whereas the researcher has both a phenomenological and psychological approach to the descriptions, the participant has not. Therefore, the results in this study have not undergone participant validation.

### Reflexivity

The first author is a professional psychologist who specializes in communication about serious illness. In the phenomenological reduction, he had to bracket this previous understanding to be open for unexpected meanings of breaking bad news. Since the initial analytical process *did* reveal new meanings, this bracketing procedure succeeded. In the subsequent disciplinary phase of the phenomenological reduction (Giorgi, 2009), the first author’s psychological background informed the transformation of the text. In the second stage of free imaginary variation, the second author approached the psychological units of meaning from another perspective: through her expertise in professional learning. Due to their dialogical imaginary variation from different perspectives, the authors could challenge each other’s viewpoints and turn the analysis into a synthesis.

### Ethics

This study was approved by the Swedish Ethical Review Authority (reg. no. 2015/557), and all participants provided written informed consent prior to enrollment in the study.

## Findings

Overall, the phenomenon of breaking bad news appears to be a *relational process* shaped by five essential interrelated meanings in the physicians’ experience. Four of these meanings were temporally constituted along the process: *Becoming the bad messenger*, *Expecting the unpredictable*, *Being on stage*, and *Professionally managing hope*. In turn, these temporal meanings were spatially constituted by a common fifth meaning, which shaped the entire process as a relational phenomenon: *Mindfulness of the emotional relationship*. Below, these essential meanings are first described individually before we synthesize and illustrate the phenomenological structure of breaking bad news in its temporal and spatial complexity.

### Becoming the Bad Messenger

Telling a patient about a severe diagnosis in a good way was generally experienced as difficult. However, in all cases, the physicians felt a responsibility to inform patients about their diagnoses as gently as possible. Yet, they had learned that no matter what words were used, they would still become the bad messenger:

Her … expression on her face changed when I said it, “for now.” I understood that I said the unmentionable even though I did not mention death. And I think that’s what made her have some kind of panic attack … after we closed the door. (P9)

While all physicians perceived themselves as the bad messenger, they had different approaches to this role. Many of them were reluctant to initiate a conversation about the diagnosis, since they empathically felt it would be painful for their patients. Yet, they had accepted it as part of their job, as P13 explained: “I imagine that’s a terrible message to receive. It’s something that has to be done anyway, and it’s my job to do it.”

Others pointed out that there was no right moment to deliver bad news, since this was only identifiable in hindsight. Therefore, they felt it was better to reveal the diagnosis and express sympathy right away. These physicians typically found relief in that they did not have a choice in this regard, although reaching such a state of mind was not always possible. For instance, when the patient’s celebration of birthdays or family holidays was at stake, the physicians could feel overwhelmed by their own frustration:

I have to tell her, and I have no idea what it will mean for her. It’s clearly really bad when you have metastases in the liver … She wants to be at home with her children, they were going to celebrate Midsummer the next day ... and then I felt like I almost just wanted to bang my head against the wall and go home because I was like “how can I do this, in a good way?” (P14)

Due to their critical role of becoming the bad messenger, the physicians were concerned about previously knowing the patient, having a patient’s family member present, having a separate room, having time to prepare, and having sufficient time for the conversation. However, because of circumstances out of the physicians’ control, they sometimes had to become a *cruel* messenger:

One of the most horrifying experiences I’ve had in any way, because I think it was so cruel for her in this shared room with a lot of devices … Meets a person, meaning me, whom she has never met and who tells her that: “Now we’re taking you back to the other ward. There’s no more treatment. And … we will take care of you.” ... I think she also asked: “Am I going to die?” And I think I said … “It’s getting close.” Well, I can’t remember the words, but I can remember this feeling (sighs) … [long silence] … it felt unworthy of her. (P11)

Apart from such stressful situations, some physicians still felt it was meaningful to break bad news when they performed the task well because this could make a big difference for a patient. Notwithstanding, even those who did declared a need to pause from becoming the bad messenger:

I see these conversations as very important, and therefore I feel satisfaction in having them ... but I can’t do it all the time, I have to take breaks sometimes and actually make people get healthier and feel better too. (P8)

### Expecting the Unpredictable

Even when the physicians knew the patient and the patient’s family members and had time to prepare for delivering a discouraging message, they could never be certain in advance of how the information would be received. According to the physicians, they were commonly targeted by patients and families in acting out their pain. Discouraging memories still lingered for those who had not managed to satisfyingly deliver their message because of the threatening responses:

They just became … frustrated and yes, angry, and very questioning towards me, and I got stressed. I think I had a hard time with that, because I started to doubt myself and became insecure when I was attacked like that. So … I couldn’t give that message with the kind of weight that was needed. (P19)

While some physicians worried about such situations, others thought that it was okay for patients and families to shoot the messenger because their own suffering should not be in focus. However, the physicians also shared completely different experiences, for example, of patients who showed no emotions or understanding of their diagnoses at all, which also caused a sense of insecurity because, as P7 explained, “Then you don’t know what’s going on in there, they [closed patients] are harder to reach. Of course, I’m wondering, have you heard what I said, have you understood?”

Thus, the physicians could feel relief when the patients or the patients’ family members cried; since then they knew that the listeners had understood and accepted the information, and it was possible for them to proceed with the conversation to questions and planning. In other cases, a patient could be calm while grasping the harsh reality—even though the physician had expected another reaction:

I thought that he would be more despairing or sad about it, but he took it quite well ... he didn’t show much in the case that he was sad, but it was more that he said “okay” or thought rationally that … “now we’re going to take one step at a time.” (P14)

Against this background, the physicians felt it was impossible to predict all situations that might arise. Hence, along with their responsibility to become the bad messenger, they always had to be prepared to change their plans for the conversation. Expecting the unpredictable was not easy, and therefore, some physicians continuously felt tense and unsafe.

### Being on Stage

The very moment of breaking bad news was typically experienced as a performance, where the physician had to be concentrated, alert, and present, without being too scared or nervous. Having witnessed how other physicians had announced bad news in brutal and unprofessional ways functioned as negative examples. Thinking about one such occasion, P5 said that “it affected me, and I thought ‘I will never do like this, it must never be like this’. I often think about it, that I don’t want to do it that way.”

Thus, in order to perform well, the physicians felt they had to be fully present, not only in their professional roles but most of all as “human beings.” As such, they had to take the entire context into account and adapt accordingly in a relational approach to their patients:

If you are going to interact with people, either [in] drama or music or whatever you do, you have to adapt in some way, you have to be sensitive to what is happening in the situation. And I think you have to be like that when you deliver this kind of message, because you don’t know exactly what they’re going to say, or think, or how they’re going to react ... and … I can be personal in there. Being on stage doesn’t have to mean that you have a facade or are impersonal. (P6)

Once they were “on stage” to break the bad news, many physicians felt they had to speak slower, use fewer words, and take more pauses than they ordinarily did in order to facilitate better communication. Some worried about increasing the patients’ distress if they did not live up to their expectations or if they were not able to answer all their questions, and P5 described that “I feel like they look to me to explain the world to them, and their world has just changed forever.”

In cases where a patient was overwhelmed with emotion, the physicians needed to deal with that issue before proceeding with more information. In this process, experienced physicians consciously shifted their approach from deciding “what I need to tell” to “what does the patient want to know?”. Recalling when they had been less experienced as physicians, some had instead focused on their message per se in this critical moment, which they retrospectively experienced as a failure. One of them (P2) shared that “I felt that I … reflected not so much on the patient himself but only on how to convey that message … [I] felt like … ‘bad doctor’.”

However, even physicians who felt confident in breaking bad news had a humble attitude toward their performance. After all, they knew that the outcome was still unpredictable, and P13 thought that “This time it went very well, it must be said. But I am of the firm conviction that there will be situations during my future professional life where things will not go as well.”

### Professionally Managing Hope

Part of what made breaking bad news a burden was handling the patients’ and families’ hopes—especially when the prospects were not at all good. In order to cope with their own distress in such situations, some physicians felt that they needed to adjust the information to preserve some room for hope. Preserving hope could mean transforming it from “hope for a cure” to another more realistic desire:

The challenge was to get them to change their hope from “we’re going to cure this” … to “okay, there’s not that hope, but we’re going to make it as good as possible, under these conditions” … so he [the patient] could be at home, and they agreed that no further treatment was appropriate. (P20)

In such cases, the physicians felt that they had created common ground with the patient. Yet in other cases, the physicians had to deal with the patients’ unrealistic hopes. Then the physician had to wait for the patient to face reality before moving on in their planning, as P1 explained: “Some patients think that they will defeat the odds, that they will be the one who survives, or that they will spend their last years of life checking off items on their bucket-list, but I know what lies ahead.”

Nonetheless, most patients’ hopes were mitigated by their bodily sensations, while the patient’s family members did not have this direct experience in their own bodies. Accordingly, the physicians knew how some patients’ family members could resist the information or just did not want to keep themselves updated with a patient’s condition. Such situations could become confusing for the physicians. For instance, when a patient’s family member had seemingly accepted the information and asked relevant questions in one conversation, thereafter, in the next conversation, they had behaved as though the patient would recover and requested unrealistic treatment:

Then I heard from the oncologist that he [the patient’s husband] had asked if they could transplant the liver. And this was only maybe three weeks before the patient died and he himself was working in healthcare, and it really felt like he wasn’t really on board. (P12)

### Mindfulness of the Emotional Relationship

Above all, most physicians cared about having a good relationship with their patients as far as possible, although sometimes they found it difficult to combine this with being the bad messenger. In such cases, the physicians imagined that their patients felt they had hurt them, and this made it difficult for them to continue treating the patient. Therefore, to avoid more harm in their relationship with the patient, they instead suggested that another physician should take over:

Sometimes the trust between me and that patient is so damaged that … you just have to say: “Maybe you want to switch to another doctor … and go to one of my colleagues.” And sometimes I get such patients from my colleagues, because they [the patients] feel that you have sort of worked against them … I also think that … you might not be able to be the one who first stabs with the knife and then comforts. (P1)

However, when the relationship *did* work satisfactorily, the physician often took on the role of a fellow human being. Rather than only hearing the patient, they were willing to listen to the patient’s history and concerns. In such moments, they could care for their patients beyond the disease, as described by P13: “There is something beautiful in actually daring to listen to people’s stories … Many people find it nice to just be able to talk about their situation and to feel that someone is actually listening and taking them seriously.”

Nonetheless, such care had a price since it involved dealing with one’s own emotions—and these became even stronger when the patients reminded the physicians of themselves or someone very close to them. Some of these physicians felt that they could connect to a patient by sharing the grief. By understanding that it was not actually about their own suffering, they had accepted that they could react with grief or be frightened themselves. For instance, P8 said that “Now I don’t care so much about myself, I can understand that I get sad, I can understand that I get scared, but it’s not me who lives on with that news.”

Yet other physicians had previous experiences of becoming too engaged and thus avoided such relationships:

I don’t want to get too involved in how the patient feels and that it is difficult for her. It will be too difficult for me. I don’t think I’m cold as a person or anything, but ... it’s like a … defense mechanism. (P22)

### Breaking Bad News as a Relational Process

The essential structure of breaking bad news is a *relational process*. In its temporal dimension, this phenomenon has its origin in the physician’s decision to tell a patient about the state of affairs, which means *becoming the bad messenger*. Subsequently, the physicians arrive at a state of mind of *expecting the unpredictable*, since they cannot know the reactions to their message. Hence, their later performance is essentially experienced as *being on stage*, not just once but in a prolonged conversation together with the progress of the disease. Therefore, sooner or later, the physician must *professionally manage hope* by weighing what a patient needs to know while also leaving some space for hope (to avoid a potential mental breakdown of the patient).

The fact that the physicians are *mindful of their emotional relationships* to their patients is spatially constitutive for the phenomenon in its entire process. Physicians either become closely involved with patients in order to compassionately support them or distance themselves from patients to protect themselves from stressful emotions. Irrespective of which approach is taken, there would be no “badness” in *becoming the bad messenger* without caring about the emotional relationship. Neither would there be any concern for *expecting the unpredictable* since this meaning is “relationally” conditioned by how the patients’ and families’ potential emotional reactions could affect the physician. Also, *professionally managing hope* is essentially a matter of being mindful of the emotional relationship because of its balancing act.

**Figure 1.**
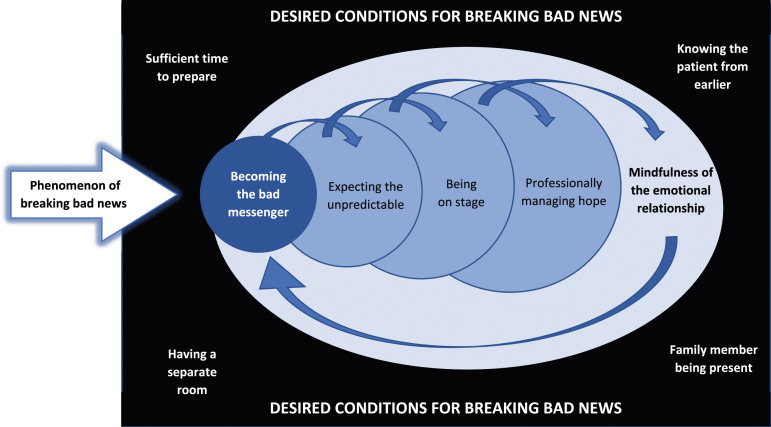
Breaking bad news as a relational process.

The essential structure of breaking bad news is illustrated in Figure 1. The black box captures the desired conditions for breaking bad news that contribute to shape a physician’s experience, while the blue circles illustrate the essential and interrelated meanings of breaking bad news. Taken together, the blue circles illustrate breaking bad news as a *relational process*, in which the upper arrows show its temporal constitution and the lower arrow shows its spatial constitution.

## Discussion

In this study, we have explored the essential structure of breaking bad news within the context of physicians’ clinical practice. While there exists much research on breaking bad news in such contexts (Bousquet et al., 2015; Matthew et al., 2019; Miller et al., 2022), our results contribute with a richer understanding of the phenomenon by revealing how breaking bad news entails more than just delivering the message and responding to emotions. More precisely, our results show the essential structure of physicians’ experiences of breaking bad news in clinical practice, including its core meanings and interrelated constitutive phases over time. Overall, this implies that their experiences of breaking bad news are fundamentally structured as a relational process.

### Effective Communication Skills Are Needed

Some physicians in our study were reluctant to deliver bad news, which is a typical human behavior (Dibble & Levine, 2010), while others found it better to just “do their job” as soon as possible. Irrespective of approach, they all had to *become the bad messenger*, which jeopardized their future relationships with the patients. One cannot think about the (subjective) delivery independently of its (intersubjective) addressee, which means that breaking bad news is fundamentally a relational act. Thus, these findings confirm that breaking news means to intersubjectively *share* serious information (Berger & Ribeiro Miller, 2022).

Furthermore, the physicians knew they had to *expect the unpredictable*, which was also stressful. This aspect is often overlooked in clinicians’ communication skills training, which focuses on preparing the patient (Johnston & Beckman, 2019) but fails to address the physicians’ own emotions in discussing patients’ prognoses and feelings (Geerse et al., 2019). Communication training in the form of, for example, learning protocols (Baile et al., 2000) and consensus guidelines for communication (Gilligan et al., 2017) cannot protect physicians from this distressing feeling. Without supplementing communication training with other affordances accordingly, physicians are still vulnerable to physiological stress reactions (Studer et al., 2017) and mental distress (Bousquet et al., 2015).

### A Complex Mindset Required on Stage

In moments of *being on stage*, many physicians felt they had to speak slower, take more pauses, and use fewer words than they normally did. The overall requirements for such performance are intricate, involving medical knowledge, self-knowledge, as well as skills of presence, receptivity, and adaptability to accommodate the patient’s immediate needs. Thus, physicians must adopt a complex mindset when breaking bad news. In our study, the physicians sometimes struggled with this complexity as they became self-conscious or task-conscious, instead of focusing on the patient—especially if they were inexperienced in executing this task. Multiple studies have shown that physicians suffer from feelings of anxiety, failure, frustration, and stress in these situations (Bousquet et al., 2015; Studer et al., 2017), which may be linked to a patient’s perception of the physician (John et al., 2019), who has to handle the complex situation.

In order to promote sustainable and compassionate providers, we believe that it would be helpful to acknowledge a wider definition of compassion as “a sensitivity to suffering *in self and others* with a commitment to try to alleviate and prevent it” (Gilbert & Simos, 2022, p. 10). One way of doing so could be to allocate sufficient time and space for serious conversations, which would make the task less painful for both physicians and patients. In medical education, physicians may then become better prepared for breaking bad news by reflecting on and discussing their own emotions and needs.

### The Importance of Understanding the Whole Process

To maintain a “therapeutic” physician–patient relationship (Miller et al., 2022), the physicians needed to compensate for being the bad messenger by delivering the news in a considerate manner while professionally managing patients’ various reactions. Continuous self-awareness and self-control were demanded of the physicians by restraining their own needs and impulses to take care of the patient, who may or may not perceive them as malicious (John et al., 2019). Given that a clinical professional attitude is reliant on accepting the unequal relationship between clinicians and patients (Holm, 2002), physicians are expected to encourage and allow the patients to express themselves within reasonable limits (Lee et al., 2002). Such professionalism could be noticed in our study, for example, in that some physicians cared for actually listening to the patients’ stories, while others thought that it was okay for patients to shoot the messenger. However, what counts as “reasonable limits” for the patients’ expression appears to be a subjective issue.

Nonetheless, the key point in our results is that breaking bad news involves concern for the continuous relationship, which confirms that breaking bad news is a prolonged process (Matthew et al., 2019; Miller et al., 2022). Also, our results illuminate the importance of physicians who understand the essential meanings and their interrelatedness in breaking bad news. Without consciousness of the dignity in becoming the bad messenger, openness in expecting the unpredictable, performative quality of being on stage, sensitivity in professionally managing hope, and essential humanity in being mindful about the emotional relationship, physicians may lack important conceptual tools that could guide them through the process of breaking bad news. Deeper understanding is thus vital to prevent an already difficult situation to be further complicated for both patients and physicians.

### Methodological Discussion

Even though the experiences of 22 physicians cannot be generalized to the entire population of clinical staff, the phenomenological design of this study enabled knowledge that should be valid beyond the studied context (Giorgi, 2006). First, physicians form a large professional group with large internal differences in terms of education, experience, and duties. Since the participants in our study came from a wide variety of specialties, there was great variation among their statements. In that sense, our data covered a range of possible angles, which is also required in the imaginative variation which analytically leads to the essential and general structure of the phenomenon studied (Giorgi, 2009). Second, due to the comparatively large number of participants for the chosen method, it was also possible to reach analytical saturation, that is, when additional descriptions added no more new information to the results.

Since all participants had signed up for a communication course, it could be argued that there was selection bias. However, we hold that this circumstance added quality to the interviews. Considering that two-thirds of the participants had signed themselves up for the course, their pronounced interest in the topic facilitated rich data. The one-third of participants who had instead been prompted by their supervisors to participate in the course (irrespective of personal interests) contributed to fulfilling the purpose of the study (Morrow, 2005). This combination is therefore considered appropriate (Polkinghorne, 2005).

## Conclusion

The results from this study show that breaking bad news in medicine is a complex relational process beyond the communicative act per se. Yet in practice, our findings reveal that physicians were sometimes forced to break bad news without having enough time for preparation and/or conversations with the patient and without knowing the patient from earlier. Such circumstances entail more stress than necessary for both physicians and patients in a situation which is in itself emotionally difficult. Since sufficient time and continuity are keys in breaking bad news, future research should survey physicians’ working conditions to enable sustainable health care.

Medical education is still highly influenced by a biomedical model (Engel, 1980), where students learn primarily about physiology and biochemistry, rather than about the essence of health care, which is human relationships (Soklaridis et al., 2016). Thus, with respect to medical education, and continuing courses for clinical staff, our empirical framework (Figure 1) may be used as a starting point for reflecting upon and discussing breaking bad news as a relational process. Overall, we suggest the following components to be educationally emphasized: being present and adaptive, being flexible in unpredictable situations, and developing compassion toward oneself and others.
